# Structural evidence for *Arabidopsis* glutathione transferase *At*GSTF2 functioning as a transporter of small organic ligands

**DOI:** 10.1002/2211-5463.12168

**Published:** 2016-12-22

**Authors:** Laziana Ahmad, Elizabeth L. Rylott, Neil C. Bruce, Robert Edwards, Gideon Grogan

**Affiliations:** ^1^ York Structural Biology LaboratoryDepartment of ChemistryUniversity of YorkUK; ^2^ Department of BiologyCentre for Novel Agricultural ProductsUniversity of YorkUK; ^3^ School of Agriculture, Food & Rural DevelopmentNewcastle UniversityUK

**Keywords:** *Arabidopsis thaliana*, *At*GSTF2, flavonoids, glutathione transferase, ligand transport, structural biology

## Abstract

Glutathione transferases (GSTs) are involved in many processes in plant biochemistry, with their best characterised role being the detoxification of xenobiotics through their conjugation with glutathione. GSTs have also been implicated in noncatalytic roles, including the binding and transport of small heterocyclic ligands such as indole hormones, phytoalexins and flavonoids. Although evidence for ligand binding and transport has been obtained using gene deletions and ligand binding studies on purified GSTs, there has been no structural evidence for the binding of relevant ligands in noncatalytic sites. Here we provide evidence of noncatalytic ligand‐binding sites in the phi class GST from the model plant *Arabidopsis thaliana*,* At*GSTF2, revealed by X‐ray crystallography. Complexes of the *At*GSTF2 dimer were obtained with indole‐3‐aldehyde, camalexin, the flavonoid quercetrin and its non‐rhamnosylated analogue quercetin, at resolutions of 2.00, 2.77, 2.25 and 2.38 Å respectively. Two symmetry‐equivalent‐binding sites (**L1**) were identified at the periphery of the dimer, and one more (**L2**) at the dimer interface. In the complexes, indole‐3‐aldehyde and quercetrin were found at both **L1** and **L2** sites, but camalexin was found only at the **L1** sites and quercetin only at the **L2** site. Ligand binding at each site appeared to be largely determined through hydrophobic interactions. The crystallographic studies support previous conclusions made on ligand binding in noncatalytic sites by *At*GSTF2 based on isothermal calorimetry experiments (Dixon *et al*. (2011) *Biochem J* **438**, 63–70) and suggest a mode of ligand binding in GSTs commensurate with a possible role in ligand transport.

**Abbreviations**
CDNB: 1‐chloro‐2,4‐dinitrobenzene
DMSO: dimethyl sulfoxide
GST: glutathione transferase
ITC: isothermal calorimetry
LB: Luria–Bertani
PDB: Protein Data Bank
PEG: polyethylene glycol

Glutathione transferases (GSTs; E.C. 2.5.1.18) are a large group of enzymes with a major role in the detoxification of xenobiotic compounds 1, 2, 3. GSTs promote the conjugation of the tripeptide glutathione (GSH) to an electrophilic centre within an acceptor molecule by deprotonating the GSH thiol, lowering the p*K*_a_ from 8.7 to 6.2, so as to form a thiolate of high nucleophilic reactivity 4. Plant GSTs have been of particular interest in recent years 5, due to their role in detoxifying xenobiotics 6, including trinitrotoluene 7 and herbicides 8. In *Arabidopsis thaliana* (*At*), a model species for plant genetic studies, 54 soluble GSTs plus one membrane‐associated protein in eicosanoid and glutathione metabolism have been identified 9. The soluble enzymes have been classified into seven distinct groups on the basis of their sequence identity: phi (F), tau (U), theta (T), zeta (Z), lambda (L), dehydroascorbate reductase and tetrachlorohydroquinone dehalogenase 9. A number of biochemical roles have been attributed to some of these groups. For example, in the zeta class, *At*GSTZ1 has been shown to have identical roles to its human homologue (*Hs*GSTZ1) with respect to tyrosine and phenylalanine catabolism 10. Many of the other classes of GSTs have less well‐defined functions, though members of the theta, tau and phi classes exhibit GSH‐dependent peroxidase activity towards organic hydroperoxides 11.

Paradoxically, while GSTs have a conserved ability to bind GSH, the only clearly established role for GSTs demonstrated *in planta* is in anthocyanin biosynthesis, where GST‐mediated conjugation does not appear to be required. First demonstrated in the maize bronze‐2 mutant (*Zm*GSTF4) 12, this phi class GST was proposed to catalyse the conjugation of cyanidin‐3‐*O*‐glucoside with GSH. However, glutathionylated anthocyanins have not been identified in plant cells. Furthermore, a phi class GST from *Petunia*, named AN9, was also shown to be involved in anthocyanin biosynthesis, but this was not dependent on conjugating activity towards these pigments *in vitro* 12. To explain the function of these tau class GSTs in flavonoid metabolism, it has been suggested that they function as carrier proteins, facilitating sequestration of anthocyanins into the vacuole 13. In support of this hypothesis, recent studies on the cytoplasmic and tonoplast‐localised *Arabidopsis At*GSTF12 (TT19) have shown that the protein can directly bind cyanidin and cyanidin‐3‐*O*‐glucoside 14.

The *Arabidopsis* phi class *At*GSTF2 has been the subject of several ligand binding studies, following the observation that the protein bound both indole‐3‐acetic acid (IAA) and 1‐*N*‐naphthylpthalamic acid (NPA), an endogenous flavonoid regulator of auxin transport 15. It was shown that NPA competed for binding with the flavonoids quercetin and kaempferol, strongly suggesting that these ligands bound to the same site in *At*GSTF2. Later studies showed that purified recombinant *At*GSTF2 bound a range of heterocyclic compounds, including the flavonoid quercetrin (quercetin‐3‐*O*‐rhamnoside), the indoles camalexin, harmane, norharmane and indole‐3‐aldehyde and the flavin lumichrome 16. These binding interactions were not disrupted by the addition of GSH, and no conjugation to the ligands was observed. Furthermore, the binding of harmane and lumichrome caused changes in the catalytic GST activity of the enzyme towards the model substrate 1‐chloro‐2,4‐dinitrobenzene (CDNB), which was suggestive of allosteric interactions that occurred at a different site(s) to the ‘G’‐ and ‘H’‐sites used for the conjugation reaction. X‐ray crystallographic evidence for the ability of GSTs to employ a distinct ‘L’ (Ligand) site for ligand transport, separate from the GSH conjugation site, has been previously provided by crystal structures of GSTs from organisms including the parasitic worm *Schistosoma japonica* 17 and human GSTs 18, 19, 20. For example, in the human GSTO1 (hGSTO1), the dye Cibacron Blue and other ligands were found to bind in the hydrophobic ‘H’ site near, but not overlapping with, the ‘G’‐site 19. A further ‘L’‐site in hGSTO1, again distinct from the GSH site, and in which the aromatic moiety of *S*‐(4‐nitrophenacyl)glutathione was bound, was found buried more deeply within the dimer interface 20. Additionally, the ligand 4‐(nitrophenol) methanethiol, thought to be a breakdown product of *S*‐(*p*‐nitrobenzyl)‐glutathione, was reported to bind to a peripheral hydrophobic binding site in the tau class GST *Gm*GSTU4‐4 from *Glycine max* 21. Furthermore, mutagenesis studies have suggested the presence of an ‘L’‐site in a phi GST from *Zea mays* (*Zm*GSTF1) that overlapped with the ‘G’‐ and ‘H’‐site 22. Despite the *in vitro* evidence for small molecule binding by members of the phi class of GSTs in *Arabidopsis*, few other structural insights into these interactions have yet been reported. In order to obtain further insight into the ligand transport properties of plant GSTs, we now report X‐ray crystallographic studies conducted with *At*GSTF2 in the presence of a range of ligands. Three structurally distinct ligands out of six of those identified as binding partners for *At*GSTF2 in previous studies were selected for study, namely indole‐3‐aldehyde **1**, camalexin **2** and quercetrin **3** (Fig. 1). The non‐rhamnosylated derivative of quercetrin, quercetin **4**, was also used as a ligand. The results, in combination with isothermal calorimetry (ITC) studies previously reported 16, provide evidence of previously unidentified ligand‐binding sites in *At*GSTF2, knowledge of which will be important in understanding the involvement of these proteins in the binding and transport of small molecules in various plant physiological processes.

**Figure 1:**
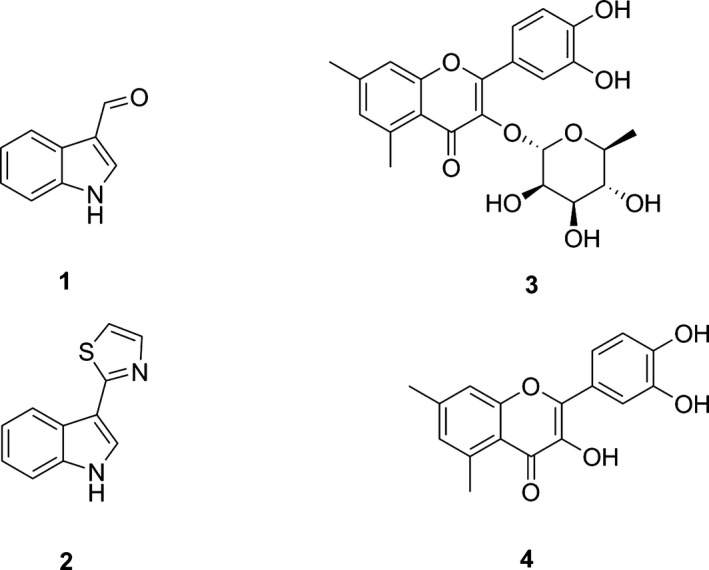
Ligands Used in this Study. **1 =** Indole‐3‐aldehyde; **2** = Camalexin; **3** = Quercetrin; **4** = Quercetin.

## Materials and methods

### Gene expression and protein purification

The pET24b vector containing the *At*GSTF2 gene, as prepared by Dixon *et al*. 16, was used to transform *Escherichia coli* Tuner (DE3) cells (Merck‐Millipore, Watford, UK) that also contained the pRARE plasmid from Rosetta (Merck‐Millipore). Transformants were grown on Luria–Bertani (LB) agar supplemented with 100 μg·mL^−1^ of kanamycin and 50 μg·mL^−1^ of chloramphenicol at 37 °C. A single colony of a plate grown overnight was used to inoculate 4 × 5 mL of LB broth. These starter cultures were grown overnight at 37 °C with shaking at 180 r.p.m. and were then used to inoculate LB broth (4 × 500 mL cultures) in which cells were grown until the optical density (OD_600_) of the culture had reached approximately 0.6. At this point, the expression of *At*GSTF2 was induced by the addition of isopropyl β‐D‐1‐thiogalactopyranoside (final concentration of 1 mm). The cultures were then incubated at 20 °C in an orbital shaker overnight at 180 r.p.m. After approximately 18‐h growth, the cells in each case were harvested by centrifugation at 4225 ***g*** for 15 min in a Sorvall RC5B Plus centrifuge (Beckman Coulter, Brea, CA, USA) and were then resuspended in Tris/HCl buffer pH 7.5 (100 mL, 20 mm, henceforth referred to as ‘buffer’). Cells were disrupted by ultrasonication for 3 × 30 s bursts at 4 °C with 1‐min intervals, and the soluble and insoluble material fractions were separated by centrifugation at 26 892 ***g*** for 30 min. The supernatant, containing the soluble *At*GSTF2, was loaded onto a 10 mL GSH sepharose 4B (GE healthcare, Chicago, IL, USA). Column fractions were analysed by SDS/PAGE and the fractions containing purified proteins were pooled and concentrated using a 10 kDa cut‐off Centricon^®^ filter membrane (Merck‐Millipore). Concentrated protein was loaded onto an S75 Superdex™ gel filtration column (GE Healthcare) that had been equilibrated with buffer also including addition of 150 mm NaCl. Fractions containing pure protein were pooled and stored at −20 °C.

### Protein crystallisation

Ligands **1**–**4** were purchased from Sigma Aldrich (Poole, Dorset, UK). Pure *At*GSTF2 was preincubated with ligands **1**,** 2**,** 3** or **4** prior to crystallisation experiments, at concentrations of either 5 or 10 mm for 1 h, followed by microcentrifugation at 16 300 ***g*** to remove any insoluble precipitates resulting from complexation. Ligand‐complexed proteins were then subjected to crystallisation trials using a Mosquito^®^ ROBOT (TTP LabTech, Cambridge, UK) and a range of commercially available crystallisation screens in 96‐well plate sitting drop format, in which each drop consisted of 150 nL protein and 150 of precipitant reservoir solution. Crystals of *At*GSTF2 in complex with indole‐3‐aldehyde **1** and camalexin **2** were obtained in 0.2 m sodium acetate and 20% (w/v) polyethylene glycol 3350. Initial crystals observed for the complex of *At*GSTF2 with quercetrin **3** and *At*GSTF2 with quercetin **4** were in 0.1 m propanoic acid, cacodylate, bis‐tris propane system and 15% (w/v) polyethylene glycol 1.5K at pH 7.0. In all cases, a protein concentration of 10 mg·mL^−1^ was employed. Larger crystals for diffraction analysis were obtained using the hanging drop vapour diffusion method in 24‐well plate Linbro dishes, with 2 μL drops consisting of a 1 : 1 ratio of mother liquor to protein solution. The best crystals of *At*GSTF2‐indole‐3‐aldehyde **1** and *At*GSTF2‐camalexin **2** complexes were obtained in drops containing 0.2 m sodium acetate and 20% (w/v) polyethylene glycol 3350 with 1% (v/v) *n*‐propanol. For *At*GSTF2‐quercetrin **3** and *At*GSTF2‐quercetin **4** complexes, the best crystals were obtained from drops using the same conditions employed in the Mosquito^®^ screen. Prior to analysis on in‐house X‐ray equipment, the crystals were washed with the mother liquor solution containing 20% (v/v) ethylene glycol as cryoprotectant and the appropriate ligand at the crystallisation concentration, followed by flash‐cooling in liquid nitrogen. Crystals were tested for diffraction using a Rigaku Micromax‐007HF fitted with Osmic multilayer optics (Sevenoaks, UK) and a MARRESEARCH MAR345 imaging plate detector (Norderstedt, Germany). Those crystals that diffracted to a resolution of equal to, or better than, 3 Å resolution were retained for data set collection at the synchrotron.

### Data collection, structure solution, model building and refinement

Complete data sets described in this report were collected at Diamond Light Source; Didcot, Oxfordshire, UK. Complexes with **1** and **2** were collected on beamline I04‐1 and complexes with **3** and **4** on beamline I03. Data were processed and integrated using xds 23 and scaled using scala 24 included in the xia2 processing system 25. Data collection statistics are given in Table 1. Complexes of *At*GSTF2‐indole‐3‐aldehyde **1**,* At*GSTF2‐quercetrin **3** and *At*GSTF2‐quercetin **4** were each in space group *P*2_1_2_1_2_1_, with six molecules in the asymmetric unit, constituting a trimer of dimers. The crystals of complex of *At*GSTF2‐camalexin **2** were in space group *P*1 with 24 molecules in the asymmetric unit, consisting of four trimers of dimers. The structure of each complex was solved using molrep 26, using a monomer of *At*GSTF2 (PDB code 1GNW; 100% sequence identity) as the model. The solvent content in the *At*GSTF2‐indole‐3‐aldehyde **1**,* At*GSTF2‐quercetrin **3** and *At*GSTF2‐quercetin **4** complexes was 42% and in the *At*GSTF2‐camalexin **2** complex was 47%. The structures were built and refined using iterative cycles using coot 27 and refmac 28, employing local NCS restraints in the refinement cycles. Following building and refinement of the protein and water molecules, clear residual density was observed in the omit maps at the dimer interfaces within the larger hexameric complexes. In each case, these could be successfully modelled as the ligands that had been used for cocrystallisation. Ligands and associated refinement libraries were prepared using prodrg 29. The complex with **1** featured three molecules of **1** per dimer, with two at the **L1** and one at the **L2** sites. The complex with **2** featured two molecules per dimer, one at each of the **L1** sites, and while there was some density at the **L2** site, the occupancy was not considered sufficiently substantial to model the camalexin ligand. The complex with quercetrin **3** featured three ligands in the dimer, at **L1** and **L2**, although, in two of the dimers, sufficient density was only observed for two ligands to be modelled. In addition, the rhamnose moiety of one of the seven ligands, at the **L2** site between subunits ‘C’ and ‘D’, could not be modelled. The complex with quercetin **4** featured one ligand per dimer, at the **L2** site in each case. The final structures exhibited % *R*_cryst_ and *R*_free_ values of 19.9/23.4 (complex with **1**); 25.0/28.4 (complex with **2**); 21.4/25.2 (complex with **3**); and 20.4/24.6 (complex with **4**). All structures were finally validated upon deposition at the PDB. Refinement statistics for all structures are presented in Table 1. The Ramachandran plot for the complex with **1** showed 98.4% of residues to be situated in the most favoured regions, 1.0% in additional allowed and 0.6% residues in outlier regions. For the complex with **2**, the corresponding values were 96.5%, 3.0% and 0.5%. For the complex with **3**, the corresponding values were 98.0%, 1.3% and 0.7%. For the complex with **4**, the corresponding values were 98.2%, 1.0% and 0.8%. Coordinates and structure factors for *At*GSTF2 complexes with indole‐3‐aldehyde **1**, camalexin **2**, quercetrin **3** and quercetin **4** have been deposited in the Protein Data Bank (PDB; http://www.rcsb.org/pdb/) with accession codes 5a4u, 5a5k, 5a4w and 5a4v respectively.

**Table 1:** Data collection and refinement statistics for *At*GSTF2 in complex with indole‐3‐aldehyde **1**, camalexin **2**, quercetrin **3** and quercetin **4**. Numbers in brackets refer to data for highest resolution shells

	Complex with indole‐3‐aldehyde **1**	Complex with camalexin **2**	Complex with quercetrin **3**	Complex with quercetin **4**
Beamline	Diamond I03	Diamond I03	Diamond I04‐1	Diamond I04‐1
Wavelength (Å)	0.97625	0.97625	0.92000	0.92000
Resolution (Å)	94.41–2.00 (2.05–2.00)	87.58–2.77 (2.84–2.77)	59.09–2.25 (2.31–2.25)	59.59–2.38 (2.44–2.38)
Space Group	*P*2_1_2_1_2_1_	*P*1	*P*2_1_2_1_2_1_	*P*2_1_2_1_2_1_
Unit cell (Å)	*a* = 87.86; *b* = 94.41; *c* = 152.38	*a* = 97.10; *b* = 113.72; *c* = 132.02	*a* = 87.35; *b* = 93.57; *c* = 152.42	*a* = 88.03; *b* = 94.83; *c* = 153.20
	α = β = γ = 90°	α = 83.7 β = 79.5 γ = 65.9°	α = β = γ = 90°	α = β = γ = 90°
No. of molecules in the asymmetric unit	6	24	6	6
Unique reflections	86 285 (6308)	126 932 (9333)	60 022 (4365)	52 158 (3838)
Completeness (%)	100.0 (100.0)	98.6 (98.1)	100.0 (100.0)	99.9 (100)
*R*_merge_ (%)	0.08 (0.63)	0.10 (0.72)	0.09 (0.72)	0.11 (0.68)
*R*_p.i.m._	0.04 (0.34)	0.10 (0.72)	0.04 (0.32)	0.07 (0.41)
Multiplicity	8.1 (8.4)	2.2 (2.2)	6.8 (7.1)	6.7 (7.0)
<*I*/σ(*I*)*>*	17.2 (3.3)	6.8 (1.8)	16.5 (3.2)	14.9 (2.8)
CC_1/2_	1.00 (0.89)	0.99 (0.74)	1.00 (0.88)	1.00 (0.84)
Overall *B* factor from Wilson plot (Å^2^)	28	35	30	21
*R*_cryst_ */R*_free_ (%)	19.9/23.4	25.0/28.4	21.4/25.2	20.4/24.6
r.m.s.d 1–2 bonds (Å)	0.017	0.014	0.012	0.012
r.m.s.d 1–3 angles (°)	1.85	1.98	1.69	1.46
Avge main chain B (Å^2^)	32	49	37	34
Avge side‐chain B (Å^2^)	35	51	40	37
Avge water B (Å^2^)	33	29	37	33
Avge ligand B (Å^2^)	26	53	51	44

## Results

### Crystal structures of *At*GSTF2 in complex with ligands 1–4

The crystal structure of *At*GSTF2 has been published previously in complex with *S*‐hexylglutathione (1GNW) 30 and also the glutathione conjugate of the herbicide FOE‐4053 (1BX9) 31. In the crystal structure 1GNW, protomers of *At*GSTF2 are found in a classical dimeric association. In order to determine crystallisation conditions for complex formation between *At*GSTF2 and the heterocyclic ligands **1**–**4**, fresh crystallisation screens were performed with the protein preincubated with 10 mm ligand (or 5 mm ligand in the case of the less soluble quercetin). Crystal complexes were obtained in each case.

The statistics for data collection and refinement are shown in Table 1. As determined with the glutathione‐conjugate complex structure 1GNW 30, *At*GSTF2 structures featured dimers in each ligand complex structure, with different numbers of monomers observed in the asymmetric unit depending on the space group. In the case of complexes formed with **1**,** 3** and **4**, crystals grew in the *P*2_1_2_1_2_1_ space group, with six monomers in the asymmetric unit. In the case of camalexin **2**, the space group was *P*1, with 24 monomers found. After building the peptide backbone, side chains and water molecules, clear residual density for each complex was observed in omit maps at an electron density level of 3σ that could be modelled as the relevant ligand in each case.

**Figure 2:**
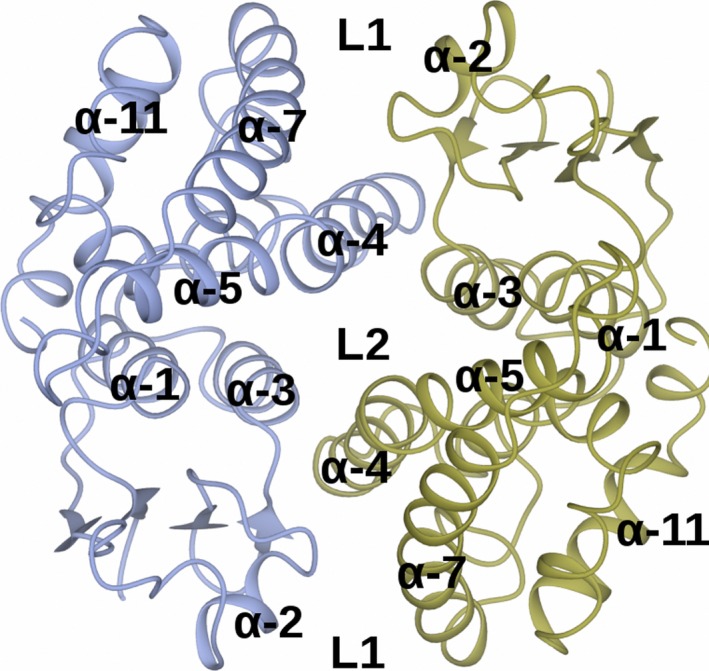
Structure of *At*GSTF2 dimer. The figure is derived using the complex with indole‐3‐aldehyde and shows selected helices and location of ligand‐binding sites **L1** and **L2** labelled for ease of reference.

**Figure 3:**
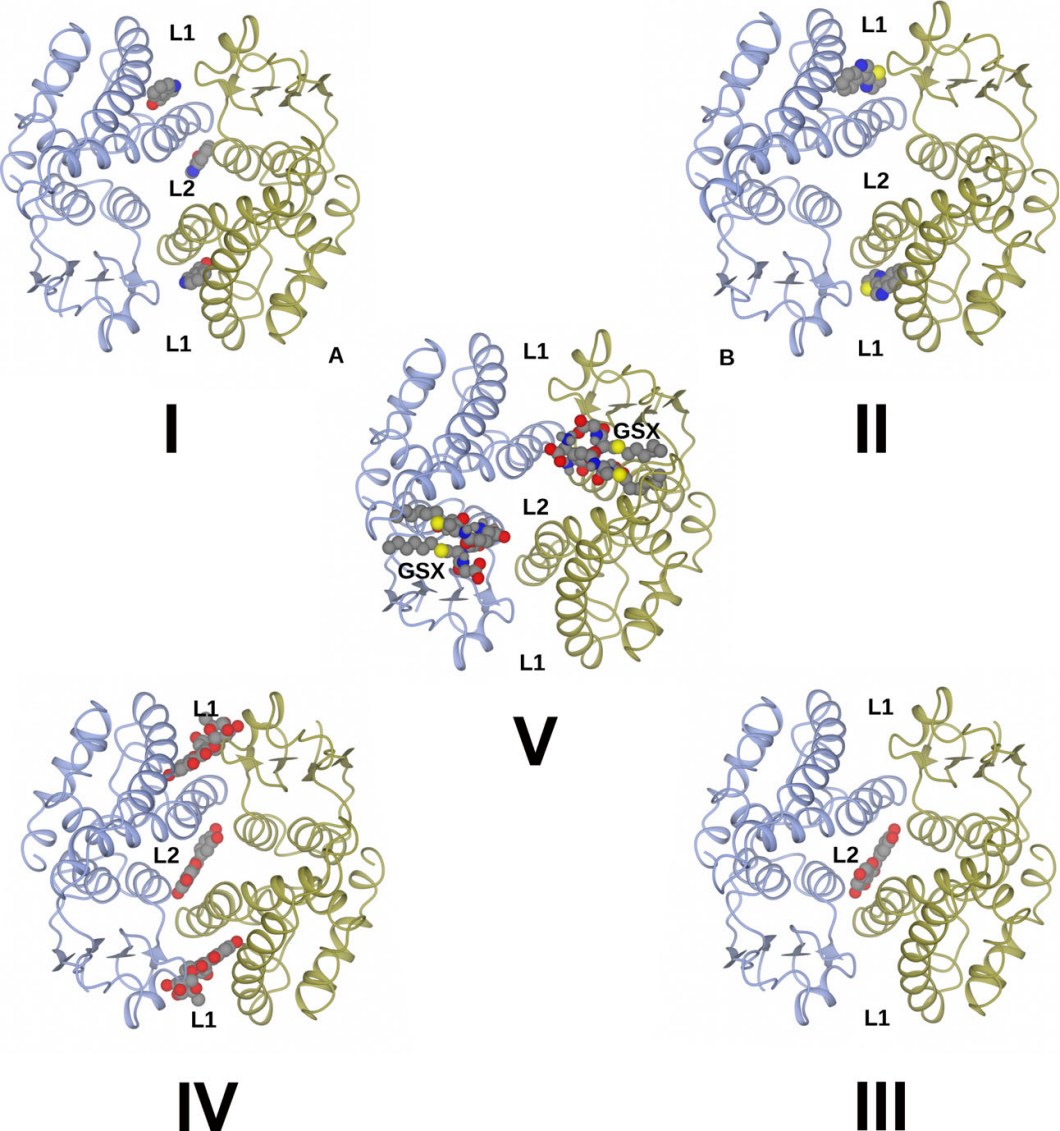
Structure of dimers ‘A/B’ from ligand complex structures of *At*GSTF2 and showing location of ligands in binding sites **L1** and **L2**. **I**: Complex with Indole‐3‐aldehyde **1**;** II**: Complex with Camalexin **2**;** III**: Complex with Quercetrin **3**;** IV**; Complex with Quercetin **4**;** V**: 1GNW, an *At*GSTF2 complex with two molecules of *S*‐hexyl glutathione ‘GSX’, showing the GSH conjugation site 30.

**Figure 4:**
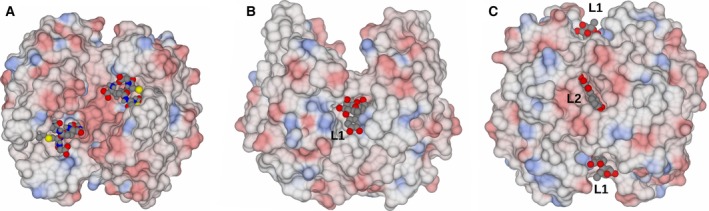
Electrostatic surface views of *At*GSTF2. (A) Same view as Fig. 3 **V**, in complex with two molecules of *S*‐hexyl glutathione (PDB code 1GNW 30); (B) In complex with quercetrin **3**, rotated 90°, and revealing ligand‐binding site **L1**; (C) In complex with quercetrin **3**, rotated 180°, and revealing ligand‐binding site **L2**.

Figure 2 shows the structure of the *At*GSTF2 dimer as observed in the complex with indole‐3‐aldehyde **1**, with the selected alpha‐helices labelled for ease of reference. Secondary structure analysis on the PDB server (http://www.rcsb.org/pdb/) shows that each monomer contains 12 α‐helices (α1: residues 13–24; α2: 46–49; α3: 68–78; α4: 94–109; α5: 112–122; α6: 124–127; α7: 134–157; α8: 168–171; α9: 174–180; α10: 186–190; α11: 193–204; and α12: 206–211), two 3_10_ helices (36–38 and 40–42) and four β‐strands (β1: 4–8; β2: 30–33; β3: 57–60; and β4: 63–66). For the complex with **1**, representative monomer pairs in a dimer superimposed with an r.m.s.d. of 0.44 Å over 209 C‐alpha atoms, with no significant differences in amino acid side‐chain positions. For complexes with **2**,** 3** and **4**, the r.m.s.d. values were 0.13, 0.37 and 0.35 Å respectively. The ligand **1** was observed in three locations in the dimer (Fig. 3I). Two of these sites, each named **L1**, were symmetry equivalent and located in a hydrophobic‐binding pocket formed between helices α‐4 and α‐7 and the loop region between Lys159 and Glu164 in each subunit of the dimer. The other site **L2** was found at the base of the dimer interface, with contributions from helix α‐3 of one monomer and α‐4 of its neighbour. None of the new ligand‐binding sites was close in space to the glutathione‐binding GSX site at the concave head of the dimer (Fig. 3V). For the complex with camalexin **2**, ligand density was only observed at the **L1** sites, with two ligands bound per dimer (Fig. 3II). By contrast, the much larger quercetrin **3**, with the pendant rhamnose, was observed at **L1** and **L2** sites in the complex structure (Fig. 3III), with three ligands in the dimer. Figure 4 illustrates surface representations of this complex. In contrast, the non‐rhamnosylated flavonol quercetin **4** only displayed ligand density in the **L2** site at the dimer interface (Fig. 3IV), with just one ligand per dimer.

### Characterisation of the ligand‐binding sites

**Figure 5:**
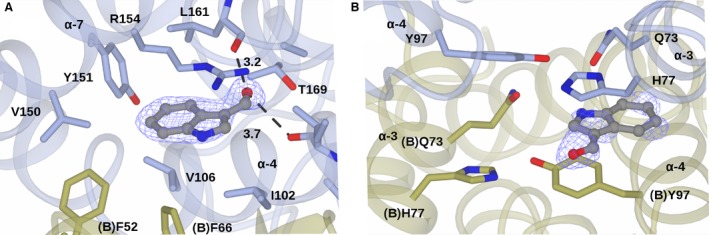
(A) Binding of indole‐3‐aldehyde **1** in the **L1** site. (B) Binding of indole‐3‐aldehyde **1** in the **L2** site. Backbone and side chains of monomers A and B of a dimer of *At*GSTF2 are shown in ribbon and cylinder format in blue and gold respectively. Indole‐3‐aldehyde **1** is shown in ball‐and‐stick format with the carbon atoms in grey. Electron density map is shown in blue and corresponds to the *F*_o_ *‐F*_c_ omit map contoured at a level of 3σ, which was obtained from refinement prior to the building of the ligand(s). Ligand atoms from the ligand complex structures have been added afterwards for clarity. Selected distances, given in Ångstroms, between protein and ligand atoms are indicated as bold dashed lines.

Detail of the ligand‐binding site **L1** from the complex with indole‐3‐aldehyde **1** is shown in Fig. 5A, with representative electron density from the relevant maps. Ligand **1** is bound in a hydrophobic pocket formed by Val150, Tyr151, Ile102, Val106 and Arg154 of one subunit (‘A’) and Phe52 and Phe66 (‘B’) of its neighbour. The plane of the aromatic ligand is sandwiched between the side chain of Arg154 and a hydrophobic shelf formed from (A)Ile102, (A)Val106 and (B)Phe52 and (B)Phe66. The aldehyde moiety is at a distance of 3.2 Å from the peptidic carbonyl of Leu161, 3.7 Å from the backbone carbonyl of Ile99, and 4.0 Å from the side‐chain hydroxyl of (A)Thr169. The indole nitrogen was not observed to make hydrogen bonding contact with any side chains in these sites.

The binding of indole‐3‐aldehyde **1** in the **L2** site was almost entirely characterised by hydrophobic interactions (Fig. 5B). The plane of the bicyclic indole is sandwiched between the side chain of (A)His77 and (B)Tyr97; the heterocyclic nitrogen is 4.1 Å from the side chain of (A)Gln73. The **L2** site is symmetrical, owing to its location at the twofold axis of the monomer interface, but ligand density for **1** was much less substantial at the putative reciprocal binding site formed by (B)His77, (A)Tyr97 and (B)Gln73, and the ligand was not successfully modelled here.

**Figure 6:**
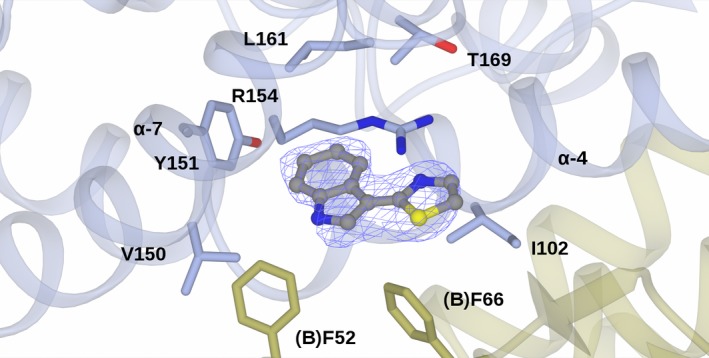
Binding of camalexin **2** in the **L1** site. Backbone and side chains of monomers A and B of a dimer of *At*GSTF2 are shown in ribbon and cylinder format in blue and gold respectively. Camalexin **2** is shown in ball‐and‐stick format with the carbon atoms in grey. Electron density map is shown in blue and corresponds to the *F*_*o*_ *‐F*_c_ omit map contoured at a level of 3σ, which was obtained from refinement prior to the building of the ligand(s). Ligand atoms from the ligand complex structures have been added afterwards for clarity.

As with **1**, the plane of the camalexin ligand again lies within the hydrophobic pocket formed by the side chains of binding site **L1** (Fig. 6). The aromatic rings of **1** and **2** can be superimposed from their complex structures, but, being a larger ligand, the thiazole ring of **2** is observed beneath the guanidinium group of Arg154, and projects more than **1** towards the periphery of the dimer. The indole ring is rotated approximately 60° relative to the orientation observed with **1**, bringing the indole nitrogen within a distance of 4.4 Å of the backbone carbonyl of Val150.

**Figure 7:**
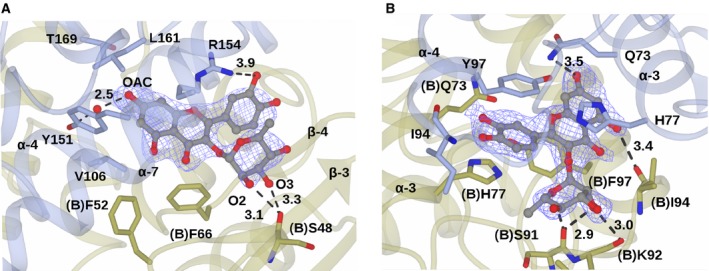
(A) Binding of quercetrin **3** in the **L1** site. (B) Binding of quercetrin **3** in the **L2** site. Backbone and side chains of monomers A and B of a dimer of *At*GSTF2 are shown in ribbon and cylinder format in blue and gold respectively. Quercetrin **3** is shown in ball‐and‐stick format with the carbon atoms in grey. Electron density map is shown in blue and corresponds to the *F*_o_ *‐F*_c_ omit contoured at a level of 3σ, which was obtained from refinement prior to the building of the ligand(s). Ligand atoms from the ligand complex structures have been added afterwards for clarity. Selected interactions between protein and ligand are indicated as bold dashed lines with distances given in Ångstroms.

The rhamnosylated flavonoid quercetrin **3** is the largest of the four ligands for which a complex was obtained. In the **L1** sites, the resorcinol ring of the flavone occupies the equivalent site to the benzene ring of **1** (Fig. 7A). The OAC hydroxyl is 2.5 Å from water molecule, which, in turn, is 2.9 Å from the phenolic hydroxyl of Tyr151. The planar bicyclic chromanone system is stacked between Arg154 and the hydrophobic shelf, as with **1**, but the side chain of Arg154 is shifted relative to the **1** complex owing to the presence of the catechol substituent on the chromanone system. The plane of the catechol ring substituent is rotated approximately 45° relative to the chromanone. The OAE catechol hydroxyl is 3.9 Å from the guanidinium group of the displaced Arg154 side chain. The rhamnose sugar assumes a conformation parallel to that of the catechol ring, with hydroxyl groups O2 and O3 3.1 and 3.3 Å, respectively, from the backbone carbonyl group of Ser48 in the ‘B’ monomer at the dimer interface.

Both quercetrin **3** and the non‐rhamnosylated quercetin **4** are found within the **L2** site. In the complex with **4**, the tricyclic flavone superimposes exactly with that of the rhamnosylated **3.** As a consequence of the twofold symmetry of this site, there is some evidence for these larger ligands being bound in reciprocal conformations. The most convincing refinement has the chromanone ring stacked between (A)His77 and (B)Tyr97. The OAC atom of the resorcinol moiety is 3.5 Å from the side‐chain amide of (A)Gln73 and the OAF at a distance of 3.4 Å from the backbone carbonyl of (B)Ile94. The catechol ring is stacked between (A)Tyr97 and (B)His77. In contrast to quercetrin binding in the **L1** site, the three rings of the flavone system are coplanar in the **L2** site (Fig. 7B). In the complex with quercetrin **3**, the rhamnose occupies a site at the periphery of the dimer, with the endocyclic oxygen 2.9 Å from the backbone carbonyl of (B)Ser91. The O2 hydroxyl of rhamnose is also 3.0 Å from the backbone carbonyl of (B)Lys92.

## Discussion

The data reported herein represent the first structures of a plant GST complexed with natural products, through selective hydrophobic interactions localised to two newly identified ligand‐binding sites **L1** and **L2**. These were remote from the active site of the enzyme more classically associated with interactions with xenobiotics, and their glutathionylated derivatives, formed following conjugation (GSX sites in Fig. 3V) 30. Each site is also distinct from the peripheral hydrophobic site previously described for 4‐(nitrophenol) methanethiol in the tau class GST *Gm*GSTU4‐4 from *G. max* 21. The residues forming the binding site **L1** do not appear to be well‐conserved among plant GSTs for which the structures have been determined, featuring neither in zeta (1E6B) 10 or tau (1GWC) 32 plant GSTs; indeed in 1GWC, a tryptophan residue W101, which superimposes with Gly103 in *At*GSTF2, occupies the **L1** site. This Trp is also conserved in the phi GST F1 (4RI6) from poplar 33. In the **L2** site, the hydrophobic residues His77 and Trp97 that form the hydrophobic pocket binding the aromatic ligands are again not conserved in 1E6B or 1GWC, being replaced by Glu and Arg residues respectively, and Asp and Lys in 4RI6.

The classes of heterocyclic ligands bound within the *At*GSTF2 structures reported herein represent important types of biologically active plant secondary metabolites derived from indoles and polyphenols respectively. The selectivity of these binding interactions is suggestive of a physiological function. Roles associated with interactions located away from the active site are most likely related to sequestration and transport of biologically active ligands. A role for these interactions in the allosteric activation of *At*GSTF2 has also been suggested, based on an observed increase in *k*_cat_ for the conjugation of the model compound CDNB with GSH in the presence of harmane 16; however, no such activation was observed for ligands **1**–**4** in the present study (data not shown) and very little change in protein structure was observed when the ligand complexes obtained herein were compared with structures of the *apo*‐protein. While we recognise the possibilities of ligand binding as a crystallographic artefact at the ligand concentrations used in this study, both binding constants and enthalpy of complex formation values determined by ITC were presented by Dixon 16, and suggest agreement with the structural observations. Quercetrin **3**, which has the most polar functionality of ligands **1**–**4**, binds in both **L1** and **L2** sites, and displays most hydrogen bonding interactions with the protein, gave a Δ*H* value of −21.1 kcal·mol^−1^ and a *K*_a_ of 0.16 μm ^−1^ in that work. Indole‐3‐aldehyde **1**, which also binds in both **L1** and **L2** sites, but makes fewer interactions, gave a less negative Δ*H* value of −13.7 kcal·mol^−1^ but a comparable value for the *K*_a_, of 0.09 μm ^−1^. Camalexin, which was observed to bind only in the **L1** site, gave the least negative Δ*H* value of −9.3 kcal·mol^−1^, and a higher affinity constant of 0.84 μm ^−1^.

The current study gives a structural basis for *At*GSTF2 being formally identified as an auxin‐binding protein 15 as well as explaining how the interactions with bioactive indoles are directly affected by competitive binding at the same L sites by specific flavonols 16, 34. Plant secondary metabolites are of great industrial importance and understanding the specificity behind GST‐ligands and how and where they are transported, would answer important biological questions, and contribute towards the genetic modification of plants for biotechnological applications.

## Author contributions

LA and ELR performed experiments; ELR, NCB, RE and GG designed experiments and supervised the work; RE and GG wrote the manuscript, with contributions from other authors.
